# A Survey of Human Gait-Based Artificial Intelligence Applications

**DOI:** 10.3389/frobt.2021.749274

**Published:** 2022-01-03

**Authors:** Elsa J. Harris, I-Hung Khoo, Emel Demircan

**Affiliations:** ^1^ Human Performance and Robotics Laboratory, Department of Mechanical and Aerospace Engineering, California State University Long Beach, Long Beach, CA, United States; ^2^ Department of Electrical Engineering, California State University Long Beach, Long Beach, CA, United States; ^3^ Department of Biomedical Engineering, California State University Long Beach, Long Beach, CA, United States

**Keywords:** review, human gait analysis, biometrics, machine learning, artificial intelligence

## Abstract

We performed an electronic database search of published works from 2012 to mid-2021 that focus on human gait studies and apply machine learning techniques. We identified six key applications of machine learning using gait data: 1) Gait analysis where analyzing techniques and certain biomechanical analysis factors are improved by utilizing artificial intelligence algorithms, 2) Health and Wellness, with applications in gait monitoring for abnormal gait detection, recognition of human activities, fall detection and sports performance, 3) Human Pose Tracking using one-person or multi-person tracking and localization systems such as OpenPose, Simultaneous Localization and Mapping (SLAM), etc., 4) Gait-based biometrics with applications in person identification, authentication, and re-identification as well as gender and age recognition 5) “Smart gait” applications ranging from smart socks, shoes, and other wearables to smart homes and smart retail stores that incorporate continuous monitoring and control systems and 6) Animation that reconstructs human motion utilizing gait data, simulation and machine learning techniques. Our goal is to provide a single broad-based survey of the applications of machine learning technology in gait analysis and identify future areas of potential study and growth. We discuss the machine learning techniques that have been used with a focus on the tasks they perform, the problems they attempt to solve, and the trade-offs they navigate.

## 1 Introduction

Smart Gait (SG) is a term for any integrated human gait data analysis system utilizing Artificial Intelligence (AI). It is a growing research field capitalizing on the advancements in modern sensing technologies, automation, cloud computing, data analytics, parallel processing, and Internet of Things (IoT).

Some of the most prominent tasks SG performs are gait phase detection, gait event prediction, human activity recognition, fall detection, recognition of a person’s age and gender, abnormal gait detection such as fatigued state, stroke and neurological disease (ND), Parkinson’s Disease (PD), estimation of joint angles and moments, the walking person’s intent recognition and trajectory prediction, human pose estimation, localization and mapping, person identification, re-identification and authentication, step counting, assessment of physical skill and mobility, balance assessment, fall risk assessment, gait modeling, and simulation. Often SG performs a combination of two or more tasks simultaneously.

SG systems can analyze single or multiple gaits simultaneously. The multi-gait SG performs tasks such as crowd and occupancy sensing, crowd behavior prediction, multi-gait recognition as in identifying a person walking with one or more others, generating multi-gait for animation and virtual environments, and detecting abnormal gait in crowds or indoors for security applications. SG is thus a smart tool in the toolbox of experts in many fields such as health and wellness, security, user privacy, forensics, enhanced user experience, animation, energy, wearables and related fields like insurance, longevity, geriatrics, workplace safety, and productivity. SG can also easily be integrated with other smart systems that utilize heart rate, audio, haptic, speech, etc., for an even wider reach across many applications and industries. As such, SG is a component of many smart devices, smart homes, stores, cities, and energy grids.

**FIGURE 1:**
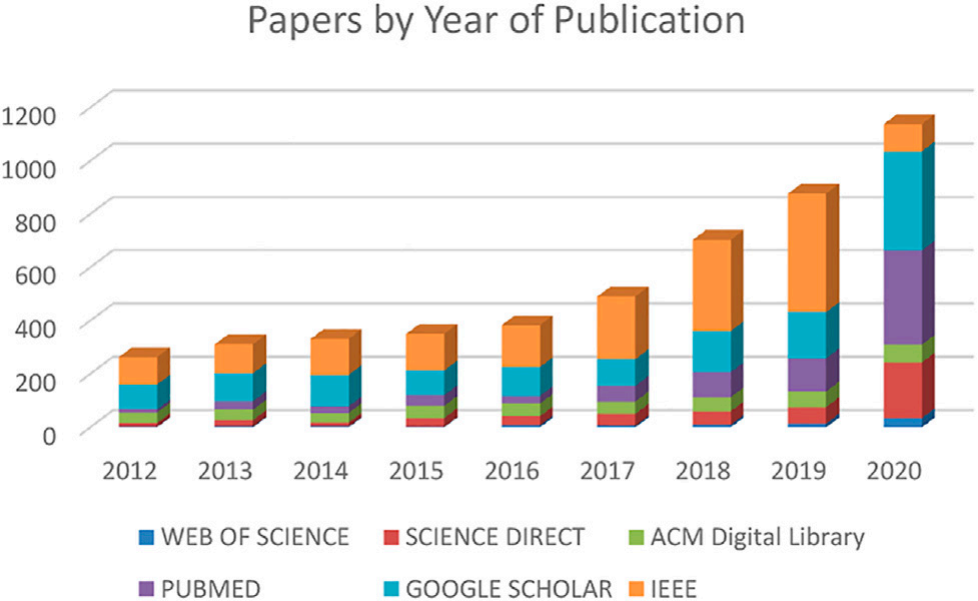
Number of papers by year of publication.

This work reviews most of the research in the field of smart gait in recent years. It provides a broad-based survey of the current state of the field and identifies future areas of potential study and growth. First, we performed an electronic database search on six well-known electronic libraries: IEEE Xplore, Science Direct, PubMed Central, Google Scholar, ACM, and Web of Science. We searched for “artificial intelligence” AND “human” AND “gait” in the full text (when available), title, keywords, and abstract. Due to the sheer volume of the work and based on the assumption that most of the more recent works build and improve upon previous work, we limited our search to 2012 to mid-year 2021. We started our work in early 2020 by reviewing papers published in 2012–2019, and updated our review with papers from 2020 and the first half of 2021 as we continued our work through the end of July 2021. All references were downloaded into EndNote. Figure 1 offers an overview of the number of references located by this original search and reflects the fast growth of SG research. Google Scholar, even though at first glance it shows the number of references in the 10,000 range, it allows downloads only up to 1,000 references. However, during our work, we came across other relevant papers that we included in this review. For instance, to identify all the work in studying fatigue by SG, we performed another search to include the word “fatigue” in our search.

Our search resulted in over 6,000 references. First, all papers were reviewed by title, and those not relevant to this study were excluded. These included papers with 1) no “gait” - such as research on upper limb movement 2) no “human” - such as research on animal and robotic gait, and 3) no AI - such as human gait studies that do not incorporate machine learning methods. After the exclusions by title, our database was reduced to about 3500 papers, which were then reviewed by title and abstract. The final selection criteria included at least one paper for each application, even smaller niche examples such as depression detection by gait (Fang et al., 2019). In areas where many papers existed for the same application, such as neurological disease gait detection, the most impactful papers were selected by looking at the overall influence of the paper by the number of citations on Google Scholar and the journal’s impact factor.

Additionally, 393 review papers were originally excluded from this study. None of the works in our database search presents a comprehensive review of the applications of AI in gait studies. These works fall in one of the three categories as below:

1) studies in related fields but not exactly within the inclusion criteria of this work, For instance, Najafi and Mishra (2021) compiled a narrative review on digital health technologies for diabetic foot syndrome. While ML algorithms are probably embedded in some of the technologies they discuss, the study does not mention or discuss artificial intelligence.
2) studies that partially overlap with our studies, such as the literature review on PD diagnosis by Mei et al. (2021), that in addition to PD diagnosis by gait, also discuss other modalities such as voice, handwriting, magnetic resonance imaging (MRI), etc.
3) studies that are in the scope of this review but only cover a specific topic such as human motion trajectory prediction (Rudenko et al., 2020), wearable sensing technologies for sports biomechanics (Taborri et al., 2020), self-powered sensors and systems (Wu et al., 2020), person re-Identification (Wang et al., 2016), (Nambiar et al., 2019), (Karanam et al., 2019), machine learning in soft robotics (Kim et al., 2021), ambient assisted living technologies (mostly AI-enabled and gait-related) (Cicirelli et al., 2021), human action recognition (Gurbuz and Amin, 2019), biomechanics (Halilaj et al., 2018), gait recognition (Kusakunniran, 2020), (Singh et al., 2018), (Wan C. et al., 2018), gait event detection and gait phase recognition (Prasanth et al., 2021), clinical gait diagnostics of knee osteoarthritis (Parisi et al., 2020), knee pathology assessment (Abid et al., 2019), data preprocessing in gait classification (Burdack et al., 2019), age estimation (Aderinola et al., 2021), and banchamrk datasets (Nunes et al., 2019). A survey paper by Alzubaidi et al. (2021) provides an overview of deep learning, with helpful definitions and a discussion of strengths, limitations, and future trends of various deep learning techniques. Similarly, Abiodun et al. (2019) review Artificial Neural Network (ANN) applications in pattern recognition. Our paper’s goal is to review applications of SG technology, in a wider, all-encompassing overview. The above-mentioned papers were not used in our study; the reader is encouraged to read them if they wish to go deeper into any of the discussed topics.

We identified six main applications of SG: 1) Gait analysis where analyzing techniques are improved through AI algorithms, 2) Health and Wellness, with applications in gait monitoring for abnormal gait detection, recognition of human activities, fall detection, and sports performance, 3) Human Pose Tracking covering one-person or multi-person tracking and localization systems, 4) Gait-based biometrics with applications in person identification, authentication, and re-identification as well as gender and race recognition 5) Smart Gait devices and environments ranging from smart socks, shoes, and other wearables, to smart homes and smart retail stores that incorporate continuous monitoring and control systems and 6) Animation that reconstructs human motion through gait modeling, simulation, and machine learning techniques. The categories are sometimes not easy to separate and overlap. For instance, gait phase detection algorithms could belong to Health and Wellness, SG Devices, or Gait Recognition categories.

The paper is organized in the following sections: Section 2) Smart Gait Vocabulary, sections 3–8 discuss the applications of AI in 3) Gait Analysis 4) Health and wellness 5) Tracking Human Pose 6) Gait Based Biometrics 7) Smart Gait devices and 8) Animation. In sections 9 and 10, we conclude with 9) Discussion and Future Trends and (10) Conclusions.

## 2 The Smart Gait Vocabulary

Here we introduce certain key definitions of essential terms commonly used in SG studies (See Table 1).

**TABLE 1:** Smart gait vocabulary.

SG Vocabulary	Definition/Context in our papers
Artificial Intelligence	Artificial Intelligence is a technology that enables computers and devices to act intelligently and make decisions like humans (Amisha et al., 2019)
Machine Learning (ML)	Machine Learning is a subfield of AI that enables computers and devices to learn from data without being explicitly programmed (Mahesh, 2020). It includes supervised learning, unsupervised learning, semi-supervised learning, and reinforcement learning. DL is a subfield of ML that extracts useful information directly from raw data to learn representations for pattern recognition (Esteva et al., 2019), (Pouyanfar et al., 2018). It is often referred to as the “black box” approach to reflect the abstract layers of human brain-like neural networks it consists of
Abnormal Gait Detection	The task of distinguishing a healthy gait from a pathological gait. Some of the pathologies that affect the walking pattern as discussed in this paper include dementia, Huntington’s disease (HD), PD, Autism Spectrum Disorder (ASD), Amyotrophic Lateral Sclerosis (ALS), Post-Stroke Hemiparetic (PSH), Acquired Brain Injury (ABI), depression, neuromuscular disease, lower extremity muscle fatigue, spastic diplegia, Cerebral Palsy (CP), etc.
Human Identification	Presented gait data is compared to a set of gait data with known identities (labeled training data) to determine whom the unknown gait belongs to
Human Re-identification	The task of identifying images of the same person from non-overlapping camera views at different times and locations. Gait is a behavioral biometric feature that is unobtrusive, hard to fake or conceal, and can be perceived from a distance without requiring the subject’s active collaboration (Nambiar et al., 2019)
Fall Detection	A binary classification task, usually concurrent with activity recognition that classifies an activity as fall or no fall
Activity Recognition	A classification task that maps features extracted from various sensor raw data to classes corresponding to activities such as sitting, lying, running, walking, stair climbing
Gender Recognition	Gender Recognition is a binary classification that maps features to qualitative outputs: male and female
Smart Home	A smart home utilizes context-aware and location-aware technologies to create intelligent automation and ubiquitous computing home environment for comfort, energy management, safety, and security (Hsu et al., 2017)
Gait Event Detection	Detection of a sequence of events that specifies the transition from one gait phase to another during each gait cycle. (Mannini et al., 2014)
Kinetic and Kinematic analysis	Kinematics studies the motion of body segments without considering masses or causal forces. Kinetics studies the relation between motion and its causes
Biometric Authentication	An automated method of verifying a person’s identity based on their biometric (gait) characteristics
Crowd Density	The density level of people in a crowded scene
Anomaly detection	It labels a behavior pattern that is "far away" from a trained model as anomalous, where “far away” is measured by a time-varying threshold (Sun et al., 2017)
Gait estimation from Pose	Parameters such as step length, stride length, stride time, cadence, etc., are estimated from the human pose
Human Gait Motion Modelling	A probabilistic manifold-based motion modeling framework able to model with a variety of walking styles from different individuals and with different strides (Ding and Fan, 2015)
Occupant Activity Sensing	Actively knowing the identity of the people within a monitored area and what they are doing (Yang et al., 2018)
Multi-Gait Recognition	Multi-gait is a term used by authors (Chen et al., 2018) to refer to the changed gait of a person walking with other people. Multi-gait recognition is the task of identifying a person when he is walking with different people
Brain-Computer Interface (BCI)	A technology that translates signals from human brain activity such as walking intention to a command sent to an external assistive, adaptive, or rehabilitative device, such as a prosthetic leg (Belkacem et al., 2020), (Khan et al., 2018)
Hybrid BCI (hBCI)	A system that fuses two bio-signals, where at least one is intentionally controlled. The different signals, such as data from electroencephalography (EEG) and functional near-infrared spectroscopy (fNIRS), are processed in real-time to establish and maintain communication between the brain and the computer. The output is evaluated through a feedback control loop. Compared with systems that use one modality alone, hBCI improves classification accuracy and the number of control commands by integrating the complementary properties of different modalities and removing artifacts (Khan et al., 2021)
Kinetic Energy Harvesting (KEH)	Technology that converts kinetic motion into energy. The individuality of the gait pattern can be captured in the output voltage signals of KEH systems, with the added benefit of energy savings, compared to accelerometers. Thus KEH systems are used as sensors and energy sources simultaneously (Lan et al., 2020) (Xu et al., 2021)
The Digital Human	Digital replicate of a human in the virtual space. Automatic, continuous gait monitoring will be an integral part of such systems (Zhang et al., 2020g)
IoT	IoT is a ubiquitous system of objects that are connected to the network, uniquely identifiable, capable of collecting, communicating, and processing data and AI-enabled to make autonomous decisions, individually or collectively (Chettri and Bera, 2019). Gait is an important biometric feature for continuous behavioral authentication in IoT systems (Liang et al., 2020)

## 3 Gait Analysis

Machine learning techniques are successfully utilized to improve many aspects of gait analysis. In this collection of papers AI 1) helps with data aggregation and pre-processing, 2) works along with another AI to improve its performance, interpretability, or accuracy, and 3) classifies gait phases and predicts gait events.

### 3.1 Data Aggregation and Pre-processing Using ML Techniques

Deep learning models can automatically extract the optimal features directly from raw spatiotemporal gait data without requiring data preprocessing or engineering (end-to-end approach) (Costilla-Reyes et al., 2021), (Morbidoni et al., 2019), etc. In other cases, when conventional machine learning techniques are deployed, much work goes into feature extraction and selection to ensure that the input features explain most of the variance in the data and achieve good performance and high accuracy of the algorithm. Of the six commonly used data pre-processing techniques, Ground Reaction Force (GRF) filtering, time derivative, time normalization, data reduction, weight normalization, and data scaling, (Burdack et al., 2020) found that only GRF filtering and supervised data reduction techniques such as Principal Component Analysis (PCA) increased the performance of ML classifiers, with Random Forest (RF) being more robust in feature reduction than Support Vector Machines (SVM), Multi-Layer Perceptron (MLP) and Convolutional Neural Networks (CNN).

PCA is a dimension reduction technique that transforms the original feature space into a set of linearly uncorrelated variables called principal components (PCs). The first few PCs alone are usually enough to account for most of the variance in the data. For instance, the first 3–6 PCs alone accounted for 84–99% of the overall variance (Dolatabadi et al., 2017). Other variants of PCA have been suggested in the SG literature, such as kernel-based PCA (Semwal et al., 2015), multi-linear PCA that achieves sparse and discriminative tensor to vector projection (Zhang et al., 2015), or a combination of PCA, linear discriminant analysis (LDA) and other feature reduction techniques (Wu C. et al., 2019). (Phinyomark et al., 2015) attempt to evaluate the importance of intermediate to higher-order PCs in running biomechanics, finding that low order PCs (that account for up to 90% of the cumulative variance in the data) can be successfully used for age and gender recognition. Still, the more subtle running behavior patterns such as between-group variations in improvements after a 6-weeks rehabilitation program of runners with patellofemoral pain (PFP) can be captured by intermediate and high order PC’s (that explain 90–99% and 99–100% of the variance of the data correspondingly). PCA, some variation of PCA, or a combination of PCA and other linear dimension reduction techniques were used in most papers we reviewed. t-Distributed Stochastic Neighbor Embedding (t-SNE) was used by Costilla-Reyes et al. (2021) to achieve a 2D representation and visualization of different age clusters of the trained data. Besides feature reduction, AI is employed for data augmentation (Bhattacharya et al., 2020) or data engineering (Johnson et al., 2021).

Other considerations in carefully selecting the input features are avoiding overfitting (Zhang et al., 2020a), improving interpretability, especially in medical applications (Dindorf et al., 2020b), (Horst et al., 2019), reducing the energy expenditure of wearable sensors (Lan et al., 2020), (Russell et al., 2021), improving patient comfort (Di Nardo et al., 2020), fairness to people with disabilities (Trewin et al., 2019) and user experience (Kim et al., 2020). To achieve the highest possible accuracy, usually, more complex sensing technology is required. The goal is to apply feature reduction and selection to avoid redundant features such that only the significant features are extracted from the minimum sensing hardware (Khademi et al., 2019). Information Gain (IG) and not PCA is used to improve interpretability by preserving and ranking the original features (Onodera et al., 2017).

### 3.2 AI2AI

We define AI2AI as an AI-based procedure whose purpose is to make another AI better. Better AI is defined based on the task. As indicated above, it includes better performance, higher accuracy, better interpretability, robustness, lower cost, lower energy expenditure, reduced overall system complexity, unobtrusive sensing technology, and automatic and real-time processing. For instance, Zhu et al. (2020) use an Improved Artificial Fish Swarm Algorithm (IAFSA) to optimize the parameters of the RF algorithm for knee contact force (KCF) prediction. Bhattacharya et al. (2020) use a novel Conditional Variational Autoencoder (CVAE) trained on an annotated dataset of 4,227 human gaits recorded on video to generate thousands of new realistic annotated gaits. Their data augmentation generative network improves the accuracy of their novel Spatial-Temporal Graph Convolutional Network (ST-GCN) algorithm to classify the four human emotions: happy, sad, angry, and neutral by 6%. Lu et al. (2021b) apply transfer learning and domain adaptation to label the data for a cross-domain human activity recognition task.

Similarly, as a first phase to creating an activity recognition algorithm for construction workers (Kim and Cho, 2020), the authors first implemented five ML algorithms 32 times each, once for every possible combination of the number of Inertial Measurement Unit (IMU) sensors and their location in the body, and compared their performance by a cross-validation (CV) technique. This systematic approach of evaluating how many sensors are sufficient for activity recognition and where they should be placed in the construction workers’ body revealed that using two motion sensors located at a certain distance will achieve motion recognition performance like using all 17 motion sensors located throughout the entire body. Using the first phase results, the authors then proceed to build a long-short term memory (LSTM) algorithm for activity recognition that achieves 94.7% accuracy with just two sensors placed in the hip and neck.

Another example of AI2AI is using AI to label the training data for another AI. Labeling data manually often requires a lot of time and expertise, is expensive, requires an elaborate lab-like setup involving obtrusive sensing technology, and must comply with safety protocols. A computer vision algorithm trained on ImageNet (He et al., 2016) labels the data with sufficient accuracy for many applications such as sports biomechanics, training, and rehabilitation (Cronin et al., 2019). Similarly, a stream-based Active Learning (AL) algorithm minimizes data labeling effort (Loy et al., 2012).

### 3.3 Gait Phase Classification and Gait Event Prediction

Gait analysis evaluates a person’s walking pattern, which is seen as a sequence of gait cycles, where each gait cycle follows the movement of a single limb from heel-strike to heel-strike again (Gage et al., 1995). The two main gait phases are the stance phase and the swing phase. Depending on the reason for gait analysis, detecting just these two phases can be enough. That simplification permits less complex and cheaper gait analysis, which is desirable, especially in wearable systems (Di Nardo et al., 2020). A more common four-phase cycle includes initial contact, mid-stance, pre-swing, and swing (Jiang et al., 2018). The importance of AI in these studies is in facilitating real-time gait analysis, appreciated in many control devices like orthotics and prosthetics, rehabilitation monitoring, and fall detection systems for aging-in-place applications. (See Table 2).

**TABLE 2:** Gait phase recognition and gait event prediction.

Reference	Algorithm	Input data	AI task
Vaith et al. (2020)	LSTM-Net, DENSE-Net	12 subjects, 7 IMUs, 2 IMU pressure insoles	offline AL to reduce labeling cost, online gait phase classification
Pérez-Ibarra et al. (2020)	Hybrid SA/GA	3 subjects, 1 HC, 2 impaired gaits, IMU at the back of the heel	online gait event detection
Di Nardo et al. (2020)	MLP	23 subjects, 1 electro-goniometer per leg	gait phase classification, 2 phases: stance/swing
Morbidoni et al. (2019)	MLP	23 healthy subjects, sEMG and barographic data, natural walking conditions	gait phase classification, 2 phases: stance/swing. Gait event prediction, HS/TO
Jiang et al. (2018)	LDA	9 healthy subjects, TW, 8 pressure sensors in an ankle-worn band	wearable gait phase recognition system
Farah et al. (2017)	DT, RF, MLP and SVM	31 subjects, an inertial sensor at the thigh	gait event detection
Taborri et al. (2014)	novel DC w/hierarchical, weighted HMMs	10 healthy subjects, TW, 2–3 IMU gyroscopes	gait phase recognition
Mannini et al. (2014)	HMM with STV	9 healthy subjects, TW, 1 IMU gyro at the instep of left foot	online gait event detection

Legend: Distributed Classifier (DC), Hidden Markov Models (HMM), Short-Time Viterbi (STV), Treadmill Walking (TW), Heel-Strike/Toe-Off (HS/TO), Simulated Annealing (SA), Genetic Algorithm (GA), Surface Electromyography (sEMG).

## 4 Health and Wellness

Clinical gait analysis, though by itself not reliable for a definitive diagnosis of neurological disease or other impairment, often suggests a pathology if it detects a pattern different from a typical walking behavior considered the normal gait. Normal gait is a controlled, coordinated, and repetitive series of limb movements that advance the body in the desired direction with minimum energy expenditure (Gage et al., 1995). There are four reasons for performing a clinical gait analysis: diagnosis, assessment, monitoring, and prediction (Baker et al., 2016). In these applications, the AI can continuously monitor and learn data, look for patterns, classify human activities and detect the anomaly. If connected to a display, it is an excellent monitoring and assessment tool. AI also predicts a future gait event, in which case it can either alert a human operator such as a clinician, caretaker, or facility supervisor or, if integrated with a control device, activate an automatic response to prevent falls or injury. For instance, WeedGait passively monitors the gait of a person and then alerts them if they are at risk of Driving Under the Influence of Marijuana (DUIM) (Li et al., 2019), while an in-home rehabilitation system provides qualitative and quantitative feedback to post-stroke survivors (Lee et al., 2019).

We identified four major applications of ML gait analysis in health and wellness: 1) detecting abnormal gait due to a person’s condition or disease, 2) sports management, 3) fall detection, and 4) activity recognition.

### 4.1 Abnormal Gait

AI is well suited at learning patterns and detecting an anomaly in the data based on a pre-defined abnormal event (supervised learning) or a clustering algorithm (unsupervised learning), or a combination of the two. A very wide range of human diseases and conditions can affect the way a person walks such as Parkinson’s (Flagg et al., 2021), (Wahid et al., 2015), Huntington’s (Acosta-Escalante et al., 2018), ALS (Aich et al., 2018), idiopathic normal-pressure hydrocephalus (iNPH) (Ishikawa et al., 2019), ASD (Hasan et al., 2018) neuromuscular disease (Gotlin et al., 2018), pediatric hereditary spastic paraplegia (HSP) (Pulido-Valdeolivas et al., 2018), aging (Strath et al., 2015), (Costilla-Reyes et al., 2021), dementia (Kenney et al., 2018), (Arifoglu and Bouchachia, 2017), fatigue (Zhang J. et al., 2014), depression (Fang et al., 2019), anxiety (Zhao et al., 2019), emotional state (Bhattacharya et al., 2020), dual task, or walking while performing a cognitive task (Costilla-Reyes et al., 2021), knee osteoarthritis, (Kotti et al., 2017), stroke (PSH gait), (Cui et al., 2018), (Clark et al., 2015), diabetes (Sutkowska et al., 2019), COVID-19 (Maghded et al., 2020), inflammation (Lasselin et al., 2020), (Renner et al., 2021), level of physical activity (Renner et al., 2021), kidney disease (Yadollahpour et al., 2018), vertigo (Cao et al., 2021), sleep quality (Liu X. et al., 2019), Trendelenburg (Michalopoulos et al., 2016), arthritis (Karg et al., 2015), (Struss et al., 2018), idiopathic toe walking (Kim et al., 2019), drunkenness (Arnold et al., 2015) and influence of marijuana (Li et al., 2019). Thus, monitoring human gait can provide key insight into a person’s health. Gait data can serve as a biomarker by itself or in association with other biomarkers, demographic data, other measured or calculated body and health parameters (Andersson et al., 2014), and patient-generated health data (PGHD) (Jim et al., 2020).

The main role of ML techniques for this cluster of studies is in classifying 1) healthy control from pathology or 2) healthy control from multiple classes of functional gait disorders. Some frequently used words in these papers, such as unobtrusive, plug-and-play system, automatic, affordable, integrated into the home or clinic, adaptable, etc., shed light on the perceived values of these applied systems and where most of the research is focused.

The benefits of utilizing machine learning in abnormal gait detection for health applications are as follows:

1) Deep learning models can automatically extract the optimal features directly from raw spatiotemporal gait data without the need for data preprocessing or engineering. (Costilla-Reyes et al., 2021).
2) A combination of ML techniques can deploy simultaneously to perform more than one task automatically. For instance, ML can simultaneously detect pathological gait and automatically identify which body part has been affected the most by the disease (Dolatabadi et al., 2017).
3) ML can process a lot of data fast, including active online learning from streams of live and repository data (Flagg et al., 2021).
4) ML community can benefit from the collaborative effort. It fosters competition against established benchmarks and open research promoted by initiatives such as the Parkinson’s Disease Digital Biomarker (PDDB) DREAM Challenge (Zhang et al., 2020a)
5) ML-driven gait-based studies can benefit from large-scale population data through smartphones, smartwatches, and other wearable devices (Frey et al., 2019), as well as individual or small-scale data easily collected at low cost, via pervasive techniques such as MS Kinect (Dolatabadi et al., 2017), smartphone (Pepa et al., 2020), triboelectric nanogenerator (TENG) smart shoes (Zou et al., 2020) and socks (Zhang et al., 2020g) which makes them suitable for implementation at home, clinic, etc. These systems stand to benefit from the recent fast and continuing advancements in sensing technology, textiles, parallel processing, cloud computing, and IoT grids.

From a practical viewpoint, using the analogy of a human operator driving a car, some common issues with these studies are:

1) Can’t drive faster than sight: the problem of data labeling.

The accuracy of the algorithm is prone to human error during the training stage. For example, to label the training data in fatigue/non-fatigue states, subjective self-reported thresholds were used for the participants in a study (Zhang J. et al., 2014). Still, the participant’s perception of fatigue is not necessarily aligned with the physical changes and walking patterns due to fatigue (Baghdadi et al., 2021). Even in stricter clinical settings where experts manually label the data, there is the problem of changed gait due to the controlled environment. Researchers usually adopt measures to mitigate these issues to some degree by studying normal walking behavior versus treadmill walking (Morbidoni et al., 2019), discarding the first few gait strides from the walking test data, instructing participants to watch a smiley on the wall to get distracted from targeting the force plate at the expense of a natural walking behavior (Horst et al., 2019) or hiding the floor sensors under the walking surface (Slijepcevic et al., 2020).

There is a need to minimize the distance between the experimental setup and the real-life application of a system. For instance, the use of a home monitoring system for abnormal gait was studied (Guo et al., 2019), but the subjects used in the study were healthy subjects imitating abnormal gaits such as in-toeing, out-toeing, drop foot, supination, and pronation. To study the gait of dementia patients, an artificially generated dataset was proposed (Arifoglu and Bouchachia, 2017) where a dataset of normal ADL data was injected with instances of skipped or repeated activities during the day and sleep disruptions at night to mimic the abnormal activities that people with dementia (PwD) would manifest. A biped that imitates human motion was used to train the data for the recognition of anterior cruciate ligament (ACL) injury of the knee (Zeng et al., 2020). Often, studies are performed on normal gait subjects and cannot be extended to a pathological gait. Trewin et al. (2019) discuss considerations of fairness for people with disabilities and outline a few guideposts on problem scoping, data sourcing and pre-processing, AI model training, and deployment. They advocate inclusive, participatory, and value-sensitive design.

Similarly, there is also the problem of a small sample size. Given the clinical nature of these studies and the impaired gait participants they require, the barriers to experimentally collecting sufficient data are understandable. For instance, 19 healthy participants had to consent to intravenous injection of lipopolysaccharide to induce inflammation in a fatigued gait study and were paid 3500SEK each (Lasselin et al., 2020). In some scenarios, the number of input features is larger than the sample size (Zhang et al., 2015). In others, the sample size is too small to be representative enough to support any assertions fully and reduces the paper to the level of an exploratory effort (Baghdadi et al., 2021). In others, researchers generate synthetic data (Arifoglu and Bouchachia, 2017) that reflect features similar to the disease for an abnormal gait detection task; apply deep AL technique to reduce the number of required labels and consequently the time cost of manual labeling in a gait phase detection task. Finally, there is a developing trend to take measurements out of the lab (Russell et al., 2021) and into the subjects’ natural environments and implement deep learning techniques to do the labeling (Costilla-Reyes et al., 2021), (Cronin et al., 2019).

Many of these techniques in aggregating, pre-processing, and learning the data, often represent work-around strategies to cost, user privacy, and clinical constraints. They both simplify the systems and introduce some errors in them simultaneously.

2) Can’t see in the dark: The Black Box problem.

This refers to the low interpretability of complex AI systems which can pose a problem, especially in medical applications. Explainable AI (XAI) is a recent trend in AI research that attempts to address this concern and the related issues of transparency, trustworthiness, and clinical acceptance (Dindorf et al., 2020b), (Khodabandehloo et al., 2021). Interpretable deep gait is the first attempt to make deep learning gait analysis more interpretable using layer-wise relevance propagation (LPR) while still achieving high accuracy (Horst et al., 2019).

3) Can’t leave the parking lot: the research to commercialization gap and the need for government approval.

Medical devices that utilize data and ML techniques will need Food and Drug Administration (FDA) approval and general buy-in from medical professionals and their patients. Doctors, clinicians, therapists, carers, et al. will need to be willing and capable of embracing the newness of the technology. To date, the authors are not aware of any FDA-approved medical devices that utilize AI and gait data. Neurodegenerative diseases are not symptomatic until years after their onset, but clinical usefulness needs to be demonstrated for the approval of a medical device. The cost of developing, installing, and maintaining such systems also becomes a barrier to their commercialization and practical usefulness (König et al., 2015).

The most recent advancements seem to address some of these concerns, but those are only in the beginning phases, and the long-term implications on user safety and privacy, as well as their actual performance, remain to be proved.

#### 4.1.1 Fatigued Gait

Fatigue is defined as “a lower level of strength, physical capacity, and performance” (Lu et al., 2017). Detecting the onset of fatigue and creating systems that manage the associated risks is an important part of production quality and human factors engineering in the workplace.

Measuring and analyzing gait for fatigue monitoring makes sense because 1) walking is a task that is possible to track via unintrusive technology suitable to workplace settings, such as video, wearable sensors, radar, and force plates on the floor or any combination of these. 2) Walking is a significant part of occupational tasks for workers in manufacturing, mining, construction, nursing, warehouse and distribution centers (Baghdadi et al., 2021). 3) in the context of advanced manufacturing (known as *Industry 4.0*) for example (but other modern occupational settings as well), in which a worker’s daily tasks involve interacting with automation, computing, and sensing technologies, the most relevant features extracted from readily available gait data can be pre-processed and analyzed through ML techniques, often real-time, which makes it possible to detect the onset of fatigue with accuracy and speed, at a relatively low cost. 4) Lastly, fatigue in the walking behavior is correlated to fatigue in other physical tasks, and while safety systems in the workplace should be custom-tailored to the relevant tasks, detecting fatigue in a worker’s gait could serve as an excellent general fatigue monitoring technique applicable to most industrial settings.

There are two main goals in fatigued gait studies: 1) intra-person recognition or continuous recognition of the person walking in different fatigue states 2) inter-person recognition or the recognition of fatigue in an individual. The first one answers the question: Can we still identify the person by their gait in a fatigued state? The second one answers the question: Can we recognize the onset of fatigue to avoid overtraining and injury in sports or improve worker safety in the workplace? Researchers conclude that a person’s gait pattern maintains its individuality even while manifesting situation-dependency, as is the case with fatigue (Janssen et al., 2011).

Only seven features extracted from one wearable sensor are needed for fatigue detection, with an average accuracy of greater than 0.85 (Sedighi Maman et al., 2020). Still, in the workplace, the individual fatigue detection accuracy of 0.85 may mean high misclassification rates across many subjects (Baghdadi et al., 2021), thus the authors suggest a multivariate hierarchical time series clustering algorithm using Dynamic Time Wrapping (DTW) as a dissimilarity measure. Detecting fatigue from smartphone sensors was suggested (Maghded et al., 2020) as part of a multi-modal sensing and machine learning framework to detect Covid-19 and predict its severity and outcome through an app on the user’s phone.

Overall, fatigue studies utilizing gait data and AI point to the importance of SG in managing fatigue in the workplace, sports performance management, rehabilitation exercises, reducing fall risk in the elderly, and finally, as part of an integrated system for overall health management, both at the individual level and in public health applications, as is the case with Covid-19 related studies. The fact that most studies we reviewed collected gait data from just one sensor or just the smartphone (Karvekar, 2019) shows that effort is already invested in making these systems unintrusive, cost-effective, and adaptable (see Table 3).

**TABLE 3:** Summary of fatigued gait studies.

Reference	Algorithm/best accuracy reported	How is data collected	Task
Russell et al. (2021)	CNN 97.8%	accelerometer worn around the chest, GPS watch for location tracking, 1 person	HAR: Climb Gate/Lay/Sit/Walk/Run. Variations in terrain and fatigue
Baghdadi et al. (2021)	MHTSCA with DTW as a dissimilarity measure	IMU worn at the right ankle 15 subjects	fatigue development over time
Sedighi Maman et al. (2020)	RF with BSS 85.5%	one sensor in the torso, 15 subjects	4-phase fatigue management framework in the workplace (1) detection (2) Identification (3) diagnosis: whole-body vs. localized (4) recovery
Maghded et al. (2020)	CNN/RNN	smartphone sensors, images and videos from the camera	detection of fatigue due to Covid-19
Karvekar, (2019)	2-class SVM 91%	24 subjects, smartphone attached to the shank	detection of fatigue: baseline, low, medium, and strong fatigued states
	3-class SVM 76%		
	4-class SVM 61%		
Baghdadi et al. (2018)	SVM 90%	one IMU in the ankle, 20 subjects	detection of fatigue after MMH tasks
Zhang et al. (2014a)	SVM 96%	17 subjects, IMU at sternum level	recognition of localized fatigued/non-fatigued state
Janssen et al. (2011)	SVM and SOM with PCA 98.1%	9 subjects GRFs	inter and intra-personal gait classification before, during, and after leg exhaustion

Legend: Best Subset Selection (BSS), Manual Material Handling (MMH), Multivariate Hierarchical Time Series Clustering Algorithm (MHTSCA).

#### 4.1.2 Neurological Disease

Gait-based detection and classification algorithms for disease diagnosis and monitoring are one of the major applications we saw, and Parkinsonian gait, with its many classifiable features, such as freezing of gait (FoG), shuffling steps, slow gait, gait asymmetry, etc., is the most prevalent disease in the studies (See Table 4). Researchers propose an automated, accurate, and sensor-free gait detection deep learning algorithm that depends on video recordings from pervasive devices such as smartphones, web cameras, and surveillance cameras as cheaper and more accessible alternatives to Vicon camera systems (Ajay et al., 2018). Procházka et al. (2015) move away from expensive, complex camera systems and recommend using MS Kinect image and depth sensors for synchronized data acquisition and spatial modeling of a moving person. The recommended Bayesian Classification (BC) algorithm distinguishes PD gait from healthy gait based on decision boundaries of three features: gait speed, stride length, and age, with an achieved accuracy of 94.1%. Wahid et al. (2015) contribute to the gait-based PD detection by suggesting that the spatial-temporal gait data be normalized first using multiple regression to account for the patient’s age, height, body mass, gender, and walking speed. (Wan S. et al., 2018) employ a deep MLP to analyze both movement and speech data captured through a smartphone and estimate the severity of PD. Ye et al. (2018) go a step further and propose a Neural Network (NN) combined with Fuzzy Logic (FL) approach that recognizes the gait of patients with neurodegenerative disease (ND) from normal gait. Since the motor function impairment in various NDs such as ALS, HD, and PD is caused by different factors, the particle swarm optimization (PSO) algorithm was used along with an adaptive neuro-fuzzy inference system (ANFIS) to classify the non-linear gait dynamics. Bilgin (2017) also attempts to distinguish ALS from other ND diseases and healthy patients using Discrete Wavelet Transform (DWT), LDA, and Naïve Bayesian Classifier (NBC).

**TABLE 4:** The pathological gait.

Reference	Algorithm/Best Accuracy	Data Collection/Input	Pathology/Task Output
Lu et al. (2020)	SVM with PCA 88.89%	Kinect camera with image rectification	Automatic depression detection
Khodabandehloo et al. (2021)	HealthXAI CART	Partial CASAS dataset, 192 subjects: 19 PwD, 54 MCI	Numerical score and explanation of the decline of cognitive functions of the elderly
Iosa et al. (2021)	ANN 93.9%	IMU at the waist belt, *N* = 33, HC = 17, stroke = 16	Stroke prognostic tool, able/unable to return to work
Flagg et al. (2021)	Bidirectional GRU	GaitNDD, GaitPDB. Streaming of live and historical GRFs	ND: PD, HD, ALS gait normality analysis
Costilla-Reyes et al. (2021)	Novel DNN with t-SNE F-score: 97.33%	Own dataset: UOM-GAIT-69. Tomography floor sensor raw data. *N* = 69, healthy normal/fast/dual-task	Age-related differences in healthy adults undertaking dual tasks
Zhu et al. (2020)	RF with IAFSA RMSE = 0.073	3 patients with knee replacement. Public dataset/challenge^2^	Knee joint impairment KFC prediction
Zhou et al. (2020)	Kernel PCA with SVM, RF, ANN: 90%	*N* = 239, young = 57, old healthy = 55, 127 = old-geriatric condition	Geriatric condition
Zhang et al. (2020a)	Deep CNN AUC = 0.87	DREAM PDDB Challenge	PD vs healthy gait; Large scale screening
Zeng et al. (2020)	RBF neural network with DL 95.61%	Kinematic modeling using a biped, *N* = 43 participants. Mocap, and force plates to test the model	Chronic unilateral ACL deficiency. Classify ACL-D/ACL-I knees
Pepa et al. (2020)	Novel FL Sp = 95.2%, Se = 84.9%	Smartphone data	Real time, interpretable FoG detection
Lasselin et al. (2020)	MLR	*N* = 19, lipopolysaccharide-induced inflammation. Kinect camera data	Effects of inflammation on human gait
Kaur et al. (2020)	LR, SVM, RF	4 people with MS. GRFs from instrumented treadmill	GML4MS framework, HC/MS mild and moderate classifier
Bhattacharya et al. (2020)	ST-GCN and CVAE 88%	4,277 human gaits in video and synthetic gaits by novel STEP-Gen	Emotion classification: happy, sad, angry, or neutral
Li et al. (2019)	WeedGait, by LSTM and SVM 92.1%	*N* = 10, smartphone data	assesses marijuana-induced gait impairment passively, warns against DUIM online
Guo et al. (2019)	SVM and BiLSTM	*N* = 16, light-weight telepresence robot equipped with a single RGB-D camera with no additional sensing feedback	normal, in-toeing, out-toeing, and drop-foot gait
(Zhang et al., 2019b)	ANN (a = 50) 93.5%	*N* = 200, 8-camera mocap and 3 force platforms	Gait classification for CP patients with spastic diplegia
Sato et al. (2019)	ST-ACF DTW, KNN with OpenPose	CASIA-B dataset. Frontal videos of two PD patients	Quantifying normal and Parkinsonian gait features from home movies
Fang et al. (2019)	RF 91.58%	95 graduate students. 52 score-depressed, 43 HC. Two MS Kinect cameras	Depression analysis
Acosta-Escalante et al. (2018)	Logitboost & RF 94.5% on raw data	*N* = 14, HD = 7, HC = 7. Smart phones (iPhone 5S) affixed to both ankles	HD gait classification
Ye et al. (2018)	ANFIS/PSO with LOOCV. 94.44%	64 subjects, ALS = 13, PD = 15, HD = 20, HC = 16, ND Public dataset. Force-sensitive switches are placed on subjects’ shoes.	Classification of Gait Patterns in Patients with various ND
Pulido-Valdeolivas et al. (2018)	RF with DTW	26 HSP and 33 healthy children. Optokinetic IGA system	Monitoring HSP progression and personalizing therapies
Wan et al. (2018b)	DMLP. 97.9%	*N* = 50, phone worn on the waist. Biomedical voice recordings (UCI dataset) and smartphone 3-axial acceleration	Analyze speech and movement data captured by smartphone to estimate the severity of PD
Hasan et al. (2018)	ANN, SVM with SWDA 93.3%	3D GRF data of 60 children: 30 ASD and 30 typically developing	Identifying ASD Gait
Cui et al. (2018)	SVM w/PCA 98.21%	*N* = 42, 21 post-stroke, 21 HC MT, GRF and EMG	Recognition and Assessment of PSH Gait
Ajay et al. (2018)	DT. 93.75%	49 YouTube videos of varying resolution. Video obtained through any pervasive devices	PD gait classification
Arifoglu and Bouchachia, (2017)	LSTM HAR: 96.7% AAD: 91.43%	Public dataset collected in 3 households through environmental sensors (Van Kasteren et al., 2011)	HAR and AAD for elderly people with dementia
Bilgin, (2017)	LDA, NBC. 90.93%	GaitNDD. Force-sensitive resistors. 3 ALS, 15 PD, 20 HD, and 16 HC	Classification of ALS among other ND diseases and healthy subjects
Dolatabadi et al. (2017)	GPLVM-thold and KNN-DTW F1-score > 0.94	*N* = 40, HC = 20, mobility impared = 20. Two Kinect sensors	Discriminate between healthy and pathological gait patterns because of stroke or ABI
Shetty and Rao, (2016)	SVM with Gaussian RBF 83.3%	GaitNDD. GRF measurements. *N* = 64, 15 PD, 18 HD, 13 ALS, 16 HC	Distinguish PD gait from HD, ALS, and HC
Procházka et al. (2015)	NBC 94.1%	*N* = 51, 18 PD, 18 HC - age-matched, and 15 young HC. MS Kinect Image and depth	PD diagnosis
Wahid et al. (2015)	RF with MR normalization. 92.6%	*N* = 49: PD = 23 HC = 26. 15 Reflected markers, 2 force platforms	PD diagnosis and management using normalized spatial-temporal gait

Legend: Decision Tree (DT), K-Nearest Neighbors (KNN), Center for Advanced Studies in Adaptive Systems (CASAS) (Cook et al., 2015), Classification and Regression Trees (CART), Gated Recurrent Unit (GRU), Root Mean Square Error (RMSE), Receiver-Operating Characteristic (ROC), ACL Deficient (ACL-D), ACL-intact (ACL-I), Radial Basis Function (RBF), Deterministic Learning (DL), Multivariable Linear Regression (MLR), Multiple Sclerosis (MS), Gait data-based ML framework for MS prediction (GML4MS), Linear Regression (LR), Abnormal Activity Detection (AAD), Gaussian Process (GP) Latent Variable Models (GPLVM), OpenPose (Cao et al., 2017).

Datasets: CASIA-B (Yu et al., 2006), Gait in Neurodegenerative Disease Database (GaitNDD) (Hausdorff et al., 2000), Gait in Parkinson’s Disease (GaitPDB) (Goldberger et al., 2000), CASAS (Cook et al., 2015), ^2^ https://simtk.org/projects/kneeloads, ND Public Dataset (Hausdorff et al., 2000).

One of the most encouraging recent developments in ND gait research using AI is the DREAM PDDB Challenge launched by Sage Bionetworks that promotes an open and competitive research infrastructure with large-scale data for developing digital signatures of PD. Similar challenges in computer vision, such as the ImageNet Large Scale Visual Recognition Challenge (ILSVRC) (Russakovsky et al., 2015), have shown to be conducive to tremendous growth with classification accuracy improving year after year against the previously established benchmark. The DREAM PDDB challenge utilizes the self-reported and sensor data collected through the mPower app (Bot et al., 2016) from 15,000 PD and healthy control (HC) subjects. The best performing team as of July 2021 reported Area Under the Receiver-Operating Characteristic Curve (AUROC) of 0.86 with a deep CNN algorithm that employs three spatial and temporal data augmentation techniques to deal with overfitting.

Another initiative, still in the early phases but very promising, is the Early Detection of Neurodegenerative Diseases (EDoN) project. Launched in February 2020, it is a global research initiative that has secured generous funding, prominent cross-disciplinary expertise, and UK government support (Wakefield, 2020). It is already in the first phase of collecting smartwatch data (gait data along with heart rate, sleep, navigation data, etc.) from volunteers in the Greater Boston area through a partnership with Boston University Alzheimer’s Disease Research Centre (BUADRC). The data will be used to generate a digital “fingerprint” for dementia which, in the application phase, will detect dementia 10–15 years earlier than the current clinical methods.

### 4.2 Sports

The general trend we see in sports and fitness applications of ML-based gait analysis systems is to create low-cost, adaptable, fast, easy, and scalable systems that reach the right balance of accuracy and comfort for the given application (See Table 5). In sports management and sports injury prevention, gait data is lower limb movements signals acquired from various sensing technologies: video, IMUs, force plates, etc. SG monitors walking and running activities but also water sports, basketball, and football.

**TABLE 5:** SG in sports.

Reference	Algorithm	Data Collection/Input	AI Task/Output
Taborri et al. (2021)	Linear SVM 96%	*N* = 39, inertial sensors, and optoelectronic bars	ACL risk prediction in female basketball players via LESS score
Johnson et al. (2021)	CNN, not enough accuracy	Wearable accelerometer	predict near real-time GRF/Ms from kinematic data
Nguyen et al. (2020)	CNN	7 IMU’s	Gait classification: athlete vs. foot abnormalities
Guo and Wang, (2021)	TS-DBN	Public datasets of videos KTH and UCF	HAR/sports behavior recognition
Gholami et al. (2020)	CNN	shoe-mounted accelerometer	Abnormal running kinematics Activity recognition
Cronin et al. (2019)	DeepLabCut	single GoPro camera	Markerless 2D kinematic analysis of underwater running
Kang et al. (2018)	FFT	Smartphone (unconstrained)	Detects walking, counts steps, irrespective of phone placement
Onodera et al. (2017)	ANN with IG	infrared cameras and force plates	Influence of shoe midsole resilience and upper structure on running kinematics and kinetics
Sundholm et al. (2014)	KNN with DTW	pressure sensor mat	Exercise detection and exercise count

Legend: Fast Fourier Transform (FFT), Time-Space Deep Belief Network (TS-DBN), Landing Error Score System (LESS), Ground Reaction Forces and Moments (GRF/M), DeepLabCut as in (Mathis et al., 2018).

Datasets: Royal Institute of Technology (KTH) (Jaouedi et al., 2020) and University of Central Florida (UCF) (Perera et al., 2019).

A deep learning algorithm trained with a relatively small number of labeled images was able to predict the locations of joint markers with the same accuracy as a human labeler, thus providing a low-cost system for kinematic analysis with sufficient accuracy for applications in sports biomechanics, training, coaching, and rehabilitation (Cronin et al., 2019). A kinematic and kinetic analysis aided by ML techniques was carried out to identify the effects of the shoes on the biomechanics of running. The conclusion: changes in the midsole resilience are more subject-dependent, but the changes in the upper shoe structure seem to be more subject-independent (Onodera et al., 2017). A smart exercise mat unobtrusively recognizes which exercise the subject is performing and counts the number of repetitions. It is soft, cheap, and smart, and it has an accuracy like pedometers when it comes to monitoring strength-related exercises performed on the ground. This is a great alternative to the exercise mats athletes commonly use in the gym (Sundholm et al., 2014).

SG systems in sports applications are diverse, performing data engineering (Johnson et al., 2021) and data labeling (Cronin et al., 2019), evaluating the role of the shoe structure on running biomechanics (Onodera et al., 2017), monitoring fatigue to prevent injury (Russell et al., 2021), (Zhang J. et al., 2014), counting steps (Kang et al., 2018), assessing physical autonomy and functional ability (Khan et al., 2015), articulating real-time control of an electrical muscle stimulation (EMS) device for sports training (Hassan et al., 2017), predicting and preventing injury (Taborri et al., 2021), achieving multi-player tracking, identification, and re-identification (Zhang R. et al., 2020), classifying and counting different sports activities (Sundholm et al., 2014), and recognizing and analyzing sports behavior (Guo and Wang, 2021).

Researchers voice concern over the validity of the laboratory setting-based AI models versus real-world scenario-based models in sports. CNN and LSTM perform better than SVM (previously suggested in the literature) in the football shot and pass detection task in three scenarios closer to the real-world setting. The integrity of the collected data, selected features, and evaluation method must be reconsidered once AI systems are deployed in the real world (Stoeve et al., 2021). Estimating kinematic data (that would usually be collected in a lab, using force plates) from kinetic data that is easily measured in the field using IMU sensors is the focus of the study by (Johnson et al., 2021). Further studies will be needed to translate the research done in the lab to systems that can reliably and accurately deploy in their practical, real-world setting, especially for time-sensitive applications such as preventing sports injury in near real-time after detecting a potentially harmful event. Additionally, an area of potential growth in the future will be the application of sports monitoring, injury prevention, and training optimization for people with disabilities (Rum et al., 2021).

### 4.3 Fall Detection and Human Activity Recognition

Gait analysis is beneficial in monitoring the activities of daily living (ADL) in the elderly to improve the quality of their lives and health care in their homes and outside hospitals. HAR using wearables poses four main concerns, and often there are tradeoffs to navigate: 1) Energy considerations 2) Activity recognition accuracy 3) robustness over different users and different activities 4) user experience. These SG systems recognize several ADLs such as bending, squatting, walking, lying down, rolling out of bed, and the transitions between them to detect falls, minimize the false alarms on lying down versus falling events while trying to keep these systems low cost, automatic, adaptable, and unobtrusive. To the effect of the low-cost fall detection systems, (Ma et al., 2014b) propose to extract curvature scale space (CSS) features of human silhouette from video clips recorded with an inexpensive Kinect depth camera. They find that their Extreme Learning Machine (ELM) algorithm, combined with a variable-length PSO algorithm, performs no worse than state-of-the-art systems that depend on expensive, complex multi-camera systems. The performance of the algorithm is enhanced by using calibration techniques to address issues of misplaced or misaligned sensors (Yu et al., 2018). The detection time is essential for fall prevention systems, such that a control device has enough time to respond and prevent the fall (Mori et al., 2020). Researchers have been concerned with the false alarms in automatic fall detection systems, especially in activities such as lying in bed and falling. Chelli and Pätzold (2019) report 100% fall detection accuracy without any false alarms when implementing quadratic SVM and ensemble bagged tree (EBT) algorithms on acceleration and angular velocity data from two public datasets. In an earlier paper, Hakim et al. (2017) also reported near-flawless accuracies on their SVM classifier using smartphone data.

Gait data for these studies were collected from force sensors and three-axis accelerometers concealed under intelligent tiles (Daher et al., 2017), built-in smartphone IMU sensors (Hakim et al., 2017), wearable sEMG sensors (Xi et al., 2017), wearable motion sensors (Özdemir and Barshan, 2014), or from an integrated data collection system such as inertial sensor and Bluetooth nodes data captured on a smartphone (Santoyo-Ramón et al., 2018). Sensor data from both a smartwatch and a smartphone is shown to work better than either one individually (Weiss et al., 2019). The smart home prides itself in capturing data real-time and device-free, utilizing wi-fi enabled IoT platforms (Yang et al., 2018). Finally, radar data from continuous-wave radar systems can be used for activity recognition and fall detection providing an unobtrusive solution for data collection and posing no privacy concerns (Wu Q. et al., 2015), (Seyfioğlu et al., 2018). CapSense sensing technology uses KEH capacitor voltage traces to recognize among five different activities with an accuracy of over 90% (Lan et al., 2017). In subsequent work, the accuracy was improved by using two capacitors, one in the sole of a shoe and one in front (Lan et al., 2020). The overall system energy usage was also reduced by 75% since the conventional machine learning algorithms such as NB or KNN used accumulated voltage data as input, reducing the computational load of the system. Future work will make these sensors fully self-powered and battery-free and address activity recognition accuracy and user experience issues.

In summary, SG systems have performed with increasing accuracy, minimizing false alarms, thus providing accurate, low-cost, automatic activity recognition and fall detection systems (see Table 6).

**TABLE 6:** Fall detection and human activity recognition.

Reference	AI Algorithm Best Achieved accuracy	Data Acquisition	Task
Chang et al. (2021)	HMM with OpenPose	Two cameras	Fall risk assessment. Evaluation of imbalanced gait
Shioiri et al. (2021)	SVM, 79%	micro-Doppler radar	Classification of gait differences associated with fall risk
	CNN 73%		
Lu et al. (2021b)	SOT, improved accuracy by 6%	Public HAR datasets UCI-DSADS, UCI-HAR, USC–HAD, PAMAP2	Cross-domain HAR, utilizing transfer learning from auxiliary labeled data
Mori et al. (2020)	NN	11 men, TW, induced disturbances	Predict falls caused by an unexpected disturbance in time for CD to deploy
Chelli and Pätzold, (2019)	ANN, KNN, QSVM, EBT. fall detection = 100%, false alarms = 0, ARA = 97.7%	Wearable sensors Public datasets (Anguita et al., 2013) and (Ojetola et al., 2015) that record falls, near-falls, and 7 ADL	ADL recognition. Fall detection
Kondragunta et al. (2019)	OpenPose for 2D pose estimation	Kinect images and sensor gait data from 250 subjects, 4 times, over 3 years	Estimation of Gait Parameters for Elderly Care from 3D Pose
Weiss et al. (2019)	RF, DT, KNN with K = 5. EER = 9.3 by RF. RF performs best in most of the sensor combinations	51 subjects, 18 ADL. Smartphones in right pocket and smartwatch on the dominant hand	Continuous biometrics authentication and identification on smartphones or smartwatches.
Santoyo-Ramón et al. (2018)	SVM, KNN, NB, DT. Error 14.162% by SVM.	Inertial sensors. 19 subjects at home, 3 falls and 11 ADL	Wearable Fall Detection System
Yang et al. (2018)	CSVD-NMF. 96.8% occupancy detection. 90.6% activity recognition	WiFi-enabled CSI measurements of 5 ADL	Device-Free Occupancy Sensing and activity recognition
Yu et al. (2018)	Gaussian HMM. Sensitivity of 0.992. Positive predictive value of 0.981	Own data. 200 fall events and 385 normal activities	Fall detection system
Seyfioğlu et al. (2018)	DCAE vs. CNN, SVM, AE.	micro-Doppler signatures	Radar-based activity recognition
Xi et al. (2017)	ARA = 97.35% by GK-SVM. FD: sensitivity 98.70% and specificity 98.59% by GK-FDA.	3 subjects, 7 ADL Wireless wearable sEMG sensors	Automatic activity recognition and fall detection
Daher et al. (2017)	HCM-SFS on fused GRF and accelerometer data. ARA> 90% on all 5 ADL.	Force sensors and accelerometers under intelligent tiles. 6 subjects, 5 ADL	Fall detection and ADL recognition in independent living senior apartments
Hakim et al. (2017)	SVM, NN, DT, DA. 99% by SVM.	Smart phone IMU. 8 healthy subjects, 4 fall events, 6 ADL	ADL recognition and threshold-based fall detection
Gao et al. (2017)	SVM	WiFi CSI measurements	Device-free wireless localization and activity recognition
Wu et al. (2015a)	Sparse BC+RVM.	2 falling, 6 ADL, Spectrograms from continuous-wave radar	Radar-based Fall Detection
(Wannenburg and Malekian 2017)	KNN, kStar, HMM, SVM, DTC, RF, NB LR, ANN	smartphone	Activity recognition
Ngo et al. (2015)	SVM, KNN	inertial sensor	Recognition for similar gait action classes
Semwal et al. (2015)	k-means and KNN ANN + PCA	vision and sensor-based gait data	Abnormal gait detection
Ma et al. (2014b)	Variable-length PSO+ELM. 91.15% sensitivity, 77.14% specificity, and 86.83% accuracy	10 young subjects, intentionally falling, and 6 ADL Kinect depth camera	Shape-based fall detection that is invariant to human translation, rotation, scaling and action length
Özdemir and Barshan, (2014)	KNN, LSM Over 99%	14 subjects, 20 falls, 16 ADL, 6 wearable sensors	Automated fall detection system
(Mannini and Sabatini, 2012)	HMM	wireless IMU and an optical motion analysis system	Gait phase detection and walking/jogging discrimination

Legend: Quadratic SVM (QSVM), HCM (Histogram Comparison Method), Sequential Forward Selection (SFS), Least squares method (LSM), Gaussian Kernel Fisher Discriminant Analysis (GK-FDA), Non-Negative Matrix Factorization (NMF), Class Estimated Basis Space Singular Value Decomposition (CSVD), Equal Error Rate (EER), Relevance Vector Machine (RVM), Gaussian Kernel SVM (GK-SVM), Substructural Optimal Transport (SOT), Channel State Information (CSI).

Datasets: UCI-DSADS (Anguita et al., 2012) UCI-HAR (Barshan and Yüksek, 2014), USC–HAD (Zhang and Sawchuk, 2012), PAMAP2 (Reiss and Stricker, 2012).

## 5 Tracking Human Pose

Applications of gait analysis to indoor environments include indoor positioning and localization algorithms. Wang et al. (2015) designed four schemes for indoor positioning. They found that WiFi Pseudo-Odometry integration with a combination of topology-constrained KNN and a multi-threshold Pedestrian Dead Reckoning (PDR) algorithm achieves higher accuracy with a smaller number of particles when a floor map is used. Other researchers (Tariq et al., 2017) found that RF performs best out of all the Weka ML collection algorithms using capacitive sensors for indoor person localization. Robertson et al. (2013) achieve SLAM using distortions of the local magnetic field. A foot-mounted sensor, for instance, can serve to localize a moving person or robot and generate a map of the indoor space while exploiting odometry. Low-cost 2D LiDAR has been recommended to preserve user’s privacy in human tracking (Hasan et al., 2020) (See Table 7).

**TABLE 7:** Tracking human pose.

Reference	AI Algorithm Best Achieved accuracy	Data Acquisition	Purpose
Vandersmissen et al. (2018)	Deep CNN. Error 21.54%	Low-power Radar. IDRad dataset made publicly available	Indoor PI invariant to the exact radar placement, room setup, and walking direction
Tariq et al. (2017)	Weka collection ML classifiers. 0.05 localization error. Accuracy > 93%	4 Capacitive Sensors in load mode	Indoor Person Localization
(Li et al., 2015)	Improved PDR algorithm The best achieved accuracy is within 2 m	Samsung Galaxy Note3 and Bluetooth beacons	PDR algorithm integrated with Bluetooth beacons for indoor positioning without additional infrastructure
Robertson et al. (2013)	MagSLAM Achieves a position accuracy of 9–22 cm	Foot mounted IMU sensors. Low-power radar device. No a priori map	Dynamic positioning (SLAM) of indoor pedestrians derives a multi-floor indoor map

## 6 Gait Based Biometrics

Gait is a soft biometric feature enabling the identification of people by their gait. The individuality of the gait pattern persists over time (Horst et al., 2017) and many pathologies. The main applications of gait recognition are person identification, person re-identification, person authentication, gender recognition, age estimation (Dindorf et al., 2020a), occupancy sensing (Yang et al., 2018), crowd density estimation (Zhou et al., 2018), crowd monitoring and anomaly detection for video surveillance applications (Sun et al., 2017), and multi-player tracking and identification (Zhang R. et al., 2020). We will look into the first four categories in more detail in sections 6.1 through 6.4.

### 6.1 Person Identification

Human identification is the process of determining an individual’s identity. Methods include but are not limited to those based on vision, identification cards, and biological data. Human gait-based identification is the process of determining an individual’s identity by their distinctive walking style. Person identification and re-identification through gait recognition have a growing importance in security systems and video surveillance in public spaces such as airports, banks, shopping malls, etc., since gait provides a non-invasive biometric feature. The task includes identifying a subject from a camera and matching him/her to persons in the other cameras with non-overlapping fields of view, an operation known as “tag-and-track” (Wu et al., 2015c). These gait recognition systems face challenges due to variable parameters that influence the size and quality of video and image inputs, such as camera viewpoint, lighting, occlusion, image resolution, and the subjects’ dressing and carrying conditions. For this reason, most papers we reviewed tried to address one or more of these difficulties by improving previously studied and implemented algorithms. To validate their work, authors conduct experiments to analyze the performance of their algorithms against benchmarks using public datasets. For instance, when CNN was first proposed for gait recognition (Wu et al., 2017), the authors evaluated their proposed algorithm on the CASIA-B (Yu et al., 2006), OU-ISIR (Iwama et al., 2012), and USF (Sarkar et al., 2005).

Most person identification SG systems deal with the case when gait data is extracted from video. These systems are model-based (Hu et al., 2012), (An et al., 2020), or appearance-based (Guan et al., 2012; Zhang W. et al., 2019), also called model-free. Gait Energy Image (GEI) is an average of all the human body silhouette images in one gait cycle (Han and Bhanu, 2005). It is widely used in appearance-based person identification algorithms because it allows for a simple representation of gait data, removing noise while preserving relevant gait information. It strikes a good balance between reducing computational cost and maintaining a good gait recognition rate. On the other hand, GEI is sensitive to appearance variations such as camera view angles and whether a subject is wearing a coat or carrying a bag, and it loses temporal information. There have been efforts to replace GEI with a representation that preserves more of the temporal gait information such as Chrono Gait Image (CGI) (Wang et al., 2011) or to fuse GEI features with temporal features (Liu et al., 2018). Chao et al. (2019) proposed GaitSet, which uses unordered sets of equally sized gait silhouettes as the input to a CNN architecture with set pooling, providing an effective way to preserve spatial and temporal information without the sequential constraints. argued that not all body parts contain discriminative information for gait recognition tasks, and they proposed GaitPart, a part-based and micro-motion model that preserves only the relevant part-dependent spatial features and the local short-range temporal information. An et al. (2020) acknowledges the challenges of appearance-based models and proposes a pose-based model approach for gait recognition. The gait poses are extracted from video using deep learning techniques AlphaPose (Fang et al., 2017) and OpenPose (Cao et al., 2017). Tracking the 3-D pose of walking pedestrians in video surveillance systems in cases where multiple people move together and cast a shadow or cause occlusion has also been attempted (Rogez et al., 2014).

Besides video, gait data can come from depth sensors, force plates, radar, Wi-Fi-enabled IoT devices, and IMU sensors. Radar-Id, a radar-based human identification algorithm (Cao et al., 2018), employs a CNN with architecture similar to that of AlexNet (Krizhevsky et al., 2012) to learn the necessary features from raw micro-Doppler spectrograms directly without a need to explicitly design the features. This algorithm has excellent anti-noise performance, and it can identify one person amid up to 20 other people. Similarly, Vandersmissen et al. (2018) use radar device data for indoor person identification and intruder detection. Costilla-Reyes et al. (2019) propose a footstep recognition system that can differentiate between the legitimate users (clients) and the impostor users of a biometric system from sensors on the floor. Biometric recognition by gait makes it possible to identify intruders since gait is difficult to fake. Deep CNN architectures that utilize footstep representations extracted from GRFs serve as automatic continuous person identification and verification systems with applications in security and anomaly detection at airports, workplace environments, and smart homes. Zhong and Deng (2014) propose a gait representation using accelerometer and gyroscope data invariant to sensor orientation. Haque et al. (2016) present a method for human identification when given only depth images. Zou et al. (2018) propose Auto-ID, a human identification system that collects CSI measurements data from WiFi-enabled IoT devices referred to as shapelet “signatures” of human identification. Yang et al. (2018) also use the CSI curve of the human body for occupancy sensing and activity recognition. This system is good for anomaly detection, such as identifying intruders in a smart home automatically. A person walking in a smart space equipped with RFID devices affects the radio frequency (RF) signals. The effect can be captured by Received Signal Strength Indicator (RSSI) and the phase of the RF signal and then used for person identification. A system that employs TSNet, a tag selection deep reinforcement learning algorithm, PCA for feature reduction, and an attention-based LSTM algorithm performs RFID-based gait recognition that can easily be integrated into a smart home or smart office environment (Luo et al., 2020).

Person identification studies have benefitted from the developments in computer vision, established benchmarks, and public datasets. The code proposed in the studies is often made public, encouraging continued and collaborative research (Chao et al., 2019). Recent studies employ deep learning approaches that extract gait representations directly from raw gait data and learn discriminative features for human identification (see Table 8)

**TABLE 8:** Person identification (PI).

Reference	Dataset/Input data	Proposed method for person identification
(Liu et al., 2018; Chao et al., 2019; Fan et al., 2020; Liu et al., 2021b)	CASIA-B and OU-MVLP	GaitPart: learns frame-level part spatial features and local short-range temporal features. Each body part has its own spatial-temporal representation
Zhang et al. (2020d)	CASIA-B, OULP, and OUMVLP.	Proposed a gait-specific loss function called angle center loss. It uses learned horizontal partitions of gait templates and a temporal attention model
Luo et al. (2020)	RSSI and phase features extracted from RF signals, 18 subjects	GRaaS; an RFID-based wireless gait recognition system using DRL tag selection algorithm and attention-based LSTM model
Chao et al. (2019)	CASIA-B and OU-MVLP	GaitSet: Deep set-based PI using Set Pooling to aggregate silhouettes into one set
(He et al., 2019; Song et al., 2019)	OU-ISIR, CASIA-B, and USF	Multi-task GANs learn view-specific gait feature presentations. Proposed PEI, a new multi-channel gait template
Costilla-Reyes et al. (2019)	SfootBD	Biometric Footstep Recognition using
	Ensemble of ResNet and SVM with floor-only sensor data
Martinho-Corbishley et al. (2019)	VIPER, CUHK, and TownCentre	Crowd prototyping. Age, gender, and ethnicity recognition using ResNet-152 CNN
(Sokolova and Konushin 2018)	TUM-GAID, CASIA-B, and OU-ISIR	Pose-based deep PI using WideResNet with OpenPose
Zou et al. (2018)	CSI measurements using two routers in IoT network, 20 subjects	AutoID: WiFi-Based PI using C3SL
Cao et al. (2018)	Radar micro-Doppler spectrograms, 24 subjects	RadarId: Deep CNN architecture based on raw radar micro-Doppler signatures
Vandersmissen et al. (2018)	Constructed own IDRad dataset from FMCW radar, 5 subjects	Indoor PI using Deep CNN with radar data; PI in the dark, privacy-preserving, intruder detection
Chen et al. (2018)	USF, CASIA-B, and OU-ISIR Own multi-gait image dataset from videos, 120 subjects in groups of 3	A model-based method for multi-gait recognition using the L-CRF model
Haque et al. (2016)	BIWI, IIT PAVIS, and IASLab	Depth-Based PI using a RNN/LSTM model. Suitable for PI in the dark
Wu et al. (2015b)	CASIA-B, PEC	Set-based PI using CNN, MLP, initialized with pretrained AlexNet
Alotaibi and Mahmood, (2015)	CASIA-B	Deep CNN framework for cross-view gait recognition,
Zhong and Deng, (2014)	McGill University and Osaka University gait datasets	KNN using GDI extracted from phone IMU data
Hu et al. (2013)	USF and CASIA-B	A unitary ViDP, matrix projects the gait templates into a latent space for view-invariant PI
Wang et al. (2012)	CASIA-B	1-NN using proposed CGI as a gait template that preserves temporal information

Legend: Convex Clustered Concurrent Shapelet Learning (C3SL), Latent Conditional Random Field (L-CRF), Gait Dynamics Image (GDI), View-Invariant Discriminative Projection (ViDP), Generative Adversarial Network (GAN), Period Energy Image (PEI), Radio Frequency Identification (RFID), Received Signal Strength Indicator (RSSI), Deep Reinforcement Learning (DRL), Gait Recognition as a Service (GRaaS), Frequency-Modulated Continuous-Wave (FMCW), ResNet-152 CNN (He et al., 2016), WideResNet (Zagoruyko and Komodakis, 2016).

Datasets: CASIA-B (Yu et al., 2006), OU-MVLP (Takemura et al., 2018), OU-ISIR (Iwama et al., 2012), OULP (Iwama et al., 2012), TUM-GAID (Hofmann et al., 2014), USF (Sarkar et al., 2005), PEC (Bossard et al., 2013), BIWI (Munaro et al., 2014), IIT PAVIS (Barbosa et al., 2012), and IASLab (Barbosa et al., 2012), McGill University Gait Dataset (Frank et al., 2010), Osaka University Gait Dataset (Ngo et al., 2014), SfootBD (Vera-Rodriguez et al., 2012), AlexNet (Krizhevsky et al., 2012).

### 6.2 Person Re-Identification

Human reidentification is the task of identifying images of the same person from non-overlapping camera views at different times and locations. A re-identification problem has three major components: identifying which human parts should be compared, constructing invariant features to represent those parts, and computing an appropriate similarity metric between them. (Saghafi et al., 2014). SG as a soft biometric feature allows continuous tracking and behavior analysis of a person over a large camera network for forensics, surveillance, and security applications. Traditional ReID methods usually focused on building robust feature representations of the gait and estimated the similarity between a probe and gallery image by calculating their Euclidian distances. This method faces challenges in cross-view and cross-walking conditions, such as when the gait pair is in different camera viewpoints and carrying and dressing conditions (Wu et al., 2017). Wu et al. (2015c) proposed that current classifiers be enhanced with a combination of Pose Prior (PP) algorithm and subject-discriminative feature selection algorithm to construct a view-invariant ReID system. One solution is to view the ReID task as a link probability prediction problem where each person represents an instance node in a graph structure. The ReID algorithm computes the likelihood of the link between the two (Liu H. et al., 2021). Another solution, suggested by Chtourou et al. (2021), includes an offline and an online phase. During the offline phase, an optimized GEI feature representation is constructed combining a dynamic selection of most relevant parts and a transformation of the probe or the gallery image, so the two of them have the same view before a matching score is calculated. This offline phase serves to train the Part View Transformation Model (PVTM), which will be used online to transform the gallery image to the same view as the probe image before classification.

ReID algorithms involve feature learning and metric learning. They learn gait features attributable to a person and then learn a similarity measure which should be greater if a gait pair belongs to different people than when it belongs to the same person. Song et al. (2018) introduced binary segmentation masks and region-level contrastive learning. Joint feature learning and similarity measure learning have also been attempted to perform both tasks well. The algorithm simultaneously extracts local convolutional features and enhances the discrimination capability by focusing only on distinct regions when looking for similarities between videos. It jointly learns features and similarity values for a pair or triplet of values (Wu L. et al., 2019). A deep Siamese attention architecture that consists of a conventional GRU and an attention mechanism can learn spatiotemporal representations and similarity metrics and learn to discriminate which local spatial representations are relevant (Wu L. et al., 2019). With large datasets of gait images, one faces the issue of multiple pedestrians having similar appearances. Chen et al. (2015) observed that the similarity metric was larger for images of the same pedestrian than for two different pedestrians Multiple deep metric learning utilizing multiple stacked auto-encoder networks and classification networks has been used to characterize different pedestrian images belonging to the same person based on multiple similarity probabilities (Xiong et al., 2019). These deep learning networks have integrated the feature learning and dissimilarity learning tasks of the traditional ReID systems into a unified deep neural network that learns representations robust to variations in image quality, background clutter, camera viewpoint and subjects’ carrying and dressing conditions directly from raw gait images at pixel level. (See Table 9)

**TABLE 9:** Human Re-Identification.

Reference	Dataset	Proposed method for ReID
Liu et al. (2021a)	Market1501, DukeMTMC-reID and CUHK03	PrGCN; Graph based method. Predicts the link probability of the node pair
Chtourou et al. (2021)	CASIA-B	PVTM: Transforms gallery image to the same view as the probe and uses only most informative human gait parts
Wu et al. (2019b)	iLIDS-VID, PRID 2011, and MARS.	Deep Siamese Attention Network Joint learning of spatiotemporal features and similarity metrics
Zhang et al. (2019c)	PRID 2011, iLIDS-VID, and SDU-VID	Multiple CNN networks Compact appearance representation of selected frames rather than whole sequence
Liu et al. (2019a)	MARS and iLIDS-VID	D3DNet, Deep metric learning
	Joint learning of spatiotemporal features and similarity metrics
Xiong et al. (2019)	VIPeR, CUHK01	Stacked Auto-Encoders Deep metric learning of multiple similarity probabilities
Song et al. (2018)	MARS, Market-1501 and CUHK03	MGCAM Binary segmentation mask and region-level triplet loss; Contrastive Learning
Wang et al. (2018)	VIPeR, PRID 450S, and CUHK01	Fine-tuned CNN with DM³. Matrix metric learning of discrepancy matrix instead of characteristic vector
Zhao et al. (2017)	ViPeR and CUHK01	KNN, SVM ReID by saliency learning and matching
Wu et al. (2015c)	ViPeR, ETHZ, SAIVT-SoftBio, and iLIDS MCTS	Improved RDC, RankSVM and PCCA by using pose priors, image rectification and online person-specific weights
Chen et al. (2015)	VIPeR, GRID, iLIDS MCTS, and CAVIAR4REID	RMLLC ReID as image retrieval task using relevance metric learning
Tao et al. (2015)	VIPeR and ETHZ	MCE-KISS Improved KISS metric learning by MCE and a smoothing technique
Ma et al. (2014a)	GRID and VIPeR	MtMCML; multi-task learning. Designed multiple distance metrics
Zheng et al. (2013)	ETHZ, iLIDS MCTS, and VIPeR	Ensemble RDC model. Relative Distance Comparison Learning

Legend: Probability Graph Convolutional Network (PrGCN), Dense 3D-Convolutional Network (D3DNet), Mask-guided Contrastive Attention Model (MGCAM), Discrepancy Matrix and Matrix Metric (DM³), Relevance Metric Learning with Listwise Constraints (RMLLC), Minimum Classification Error (MCE) Keep it simple and straightforward (KISS) Metric Learning, Multi-task Maximally Collapsing Metric Learning (MtMCML), Relative Distance Comparison (RDC), Support Vector Ranking (RankSVM), Pairwise Constrained Component Analysis (PCCA).

Datasets: VIPeR (Gray and Tao, 2008), CUHK01(Li et al., 2012), iLIDS-VID (Wang et al., 2014), PRID 2011 (Hirzer et al., 2011), and MARS (Zheng et al., 2016), SDU-VID (Liu et al., 2015), Market1501 (Felzenszwalb et al., 2008; Zheng et al., 2015), DukeMTMC-reID (Ristani et al., 2016), CUHK03 (Li et al., 2014), PRID 450S (Roth et al., 2014), GRID (Loy et al., 2009), iLIDS MCTS (Zheng et al., 2009), and CAVIAR4REID (Cheng et al., 2011), ETHZ (Schwartz and Davis, 2009), SAIVT-SoftBio (Bialkowski et al., 2012).

### 6.3 Person Authentication

Proper authentication includes user authentication: whether the user has authorized access, and person identification: who the current user is (Liang et al., 2020). Sensor-based user authentication uses biological features categorized in physical, physiological, and behavioral (Hernández-Álvarez et al., 2021). Gait provides behavioral biometric-based authentication, which by comparison with knowledge-based (passwords, personal identification number (PIN)) and physiological biometric-based (face recognition, fingerprint) is unobtrusive, continuous, less prone to attacks, and easily tracked through wearable devices, videos, and smartphones in the context of IoT environments. Gait-based authentication is studied either by itself (Qin et al., 2019) or as part of an authentication system that uses multiple modalities (Hintze et al., 2019) (Acien et al., 2019) (See Table 10). Authors (Acien et al., 2019) showed that the fusion with behavioral data improves the authentication system results.

**TABLE 10:** Person authentication (PA).

Reference	AI Algorithm	Dataset/Data Modality	Purpose
Zhang et al. (2020b)	SVDD and PCA for illegal user detection, LSTM for PI	Velocity and acceleration from the smartphone at the leg	PI and illegal user detection
Li et al. (2020)	Two-stream CNN with SVM	BrainRun dataaset. Own dataset of gait and other behavioral features from smartphones, 100 subjects.	SCANet: Continuous PA, distinguishes legitimate vs impostor users
(Zhang et al., 2014b; Qin et al., 2019)	Multi-layer LSTM and Extreme Value Statistic	ZJU-GaitAcc, 3D accelerations from smartphones	PI and PA of the learned user, reject unauthorized user
(Wu et al., 2018; Hintze et al., 2019)	SVM, KNN, DT	acceleration, angular velocity, magnetic intensity, and PPG signals from fingertip device	Multisensor PA, HAR
Vandersmissen et al. (2018)	Deep CNN	Own IDRad Dataset: micro-Doppler signatures, 5 subjects	Automatic intruder detection, indoor PI
Jorquera Valero et al. (2018)	Semi-supervised ML, Isolation Forest	Tracking current vs. known usage of the device and motion sensor data from phone	Adaptive and continuous PA system, anomaly detection
Neverova et al. (2016)	Dense clockwork RNN	HMOG, Google Abacus Dataset: time series of inertial measurements	distinguishes legitimate vs impostor users

Legend: Photoplethysmography (PPG), Support Vector Data Description (SVDD), Growing Neural Gas (GNG).

Datasets: BrainRun (Papamichail et al., 2019), ZJU-GaitAcc (Zhang Y. et al., 2014), HMOG (Yang et al., 2014), UMN (Raghavendra et al., 2006), UCSD Ped (Li et al., 2013), Avenue (Lu et al., 2013).

### 6.4 Gender Recognition

SG performs gender recognition reliably and unobtrusively (See Table 11). The gender classification task is usually conducted alongside human identification, and human re-identification tasks (Lu et al., 2014) since a person’s gender can serve as a soft feature in identifying a person. For instance, gender classification is done first to prune a subset of subjects before human identification is performed (Castro et al., 2017), (Meena and Sarawadekar, 2020). Gender classification is also done first to improve the accuracy of a subsequent age estimation algorithm (Zhang S. et al., 2019). Gender and age classification, in certain commercial and electronic consumer applications specifically, can be sufficient to enhance user experience (Duan et al., 2018).

**TABLE 11:** Gender recognition (GR).

Reference	AI Algorithm Best Achieved accuracy	Dataset/Input features	Task
Kwon and Lee, (2021)	KNN, SVM, NB, DT, 100%	UPCVgaitK1, UPCVgaitK2	GR
Zhang et al. (2019b)	multi-task CNN, AE: MAE = 5.47, GR: 98.1%	OULP-Age dataset GEI from video	GR and AE
El-Alfy and Binsaadoon, (2019)	LK-SVM with FLBP Normal: 96.40%	CASIA-B GEI from video	GR
	Carrying: 87.97%		
	Wearing coat: 86.54%		
(Jain and Kanhangad, 2018)	Bootstrap DT 94.44%	1D HG extracted from Smartphone in the front pocket	GR
(Castro et al., 2017)	CNN F:77%, M:96%	TUM-GAID: extracted from low-resolution video streams recorded with MS Kinect	automatic PI and GR
Lu et al. (2014)	AP clustering + SRML PI: 87.6% GR: 93.1%	Own dataset: ADSCAWD USF and CASIA-B C-AGI instead of GEI from MS Kinect Depth Sensor	PI and GR

Legend: Sparse Reconstruction-based Metric Learning (SRML), Cluster-based Averaged Gait Image (C-AGI), Affinity Propagation (AP), Optical Flow (OF), Person Identification (PI), Gender Recognition (GR), Age Estimation (AE), Fuzzy Local Binary Pattern (FLBP), Linear Kernel SVM (LK-SVM).

Datasets: UPCVgaitK1 (Kastaniotis et al., 2013), UPCVgaitK2 (Kastaniotis et al., 2016), OULP-Age (Iwama et al., 2012), CASIA-B (Yu et al., 2006), TUM-GAID (Hofmann et al., 2014), USF (Sarkar et al., 2005).

The authors have explored different input features for this task. Joint Swing Energy (JSE) is a static feature extracted from the skeleton, namely the distance of the body joints from anatomical planes. It can easily be extracted from gait data and performs well with various classifiers to recognize someone’s gender (Kwon and Lee, 2021). Histogram of Gradient (HG) method reduces the three-dimensional (3D) accelerometer and gyroscope data from smartphones into 1D temporal descriptors, used as input for a bootstrapped DT algorithm (Jain and Kanhangad, 2018). Small walking speed variations do not affect the classification accuracy, but larger variations do, suggesting that spatial features are probably better suited for gender recognition tasks using conventional classifiers. Authors (Wazzeh et al., 2019) suggested that extracting features that are invariant to walking speeds variations could improve the performance of their gender recognition algorithm. Castro et al. (2017) proposed a CNN-based end-to-end approach that uses optical flow maps extracted from very low-resolution video to represent each person by their gait signature and recognize their gender and identity with high accuracy. A multi-task CNN setup, where the deep network learns multiple attributes simultaneously, improves the accuracy further (Zhang S. et al., 2019).

Some authors focus on recognizing gender from gait data when subjects walk in different directions (Lu et al., 2014), at different walking speeds (Jain and Kanhangad, 2018), carrying a bag, wearing a coat (El-Alfy and Binsaadoon, 2019), etc. SG for gender recognition is non-intrusive, does not require the subject to cooperate, and has better performance invariant to carrying and clothing conditions, even with the low-resolution quality of videos.

## 7 Smart Gait Devices and Environments

This section reviews systems that incorporate ML, IoT, and advanced sensing and textile technologies for automatic, real-time gait data processing to perform an intelligent task with health, sports, entertainment, and security applications.

### 7.1 Smart Gait Devices

The domain of smart gait devices and environments is exciting, brave, creative, extensive, and ever-growing (See Table 12). SG devices include wearable shoes (Zou et al., 2020), socks (Zhang et al., 2020f), kneepads and anklets (Totaro et al., 2017), insoles (Low et al., 2020), as well as devices attached to the body, such as smartphones (Poniszewska-Maranda et al., 2019), smartwatches (San-Segundo et al., 2018), (Sigcha et al., 2021), etc., implantable medical devices such as ActiGait (Sturma et al., 2019), wearable robotics (Shi et al., 2019) such as prosthetics (Gao et al., 2020) orthotics (Zhang et al., 2020e), (Choo et al., 2021), assistive devices such as smart walkers (Jimenez et al., 2018), and environmental devices such as smart tiles (Daher et al., 2017). SG devices use gait data to facilitate health monitoring, including passive mental health assessment (Rabbi et al., 2011) and transfer data to control devices for health, sports, security, and entertainment applications. For instance, UbiHeld (Ubiquitous Healthcare for Elderly) incorporates gait and other data from a smartphone as well as additional data from an inexpensive Kinect camera to keep a status of the overall health, location, and activities of the elderly at home (Ghose et al., 2013).

**TABLE 12:** Smart gait devices.

Reference	AI Algorithm	AI task	Sensing Technology	Application
Yang and Yin, (2021)	LSTM with CAE	estimating joint torque for motion intent prediction	three soft pneumatic sensors two 3D IMUs	soft smart shoes
Xu et al. (2021)	attention-based LSTM	gait recognition while preserving privacy of users	KEH	PrivGait, a KEH-equipped wearable device
Sandhu et al. (2021)	RF	human activity recognition	wrist-worn solar cell	SolAR, a solar self-powered wearable device
Zhang et al. (2020g)	1D CNN	gait and human activity recognition	textile TENGs	smart socks for long-term gait monitoring
Lan et al. (2020)	NB, RF, DT, KNN	human activity recognition	two capacitors and two transducers	a self-powered shoe with embedded CapSense technology
Gao et al. (2020)	RNN	imitation learning for real-time prosthetic control	built-in motion sensors	powered transfemoral prosthesis
Zhang et al. (2020e)	RL	assist-as-needed control for robot-assisted gait training	built-in motion sensors	SAFE orthosis
Llorente-Vidrio et al. (2020)	DDNN	classification of EMG signals to activate an event-driven controller	EMG sensors	mobile lower limb active orthosis

Legend: Deep Differential Neural Networks (DNNN), Stevens Ankle-Foot Electromechanical (SAFE), Reinforcement Learning (RL).

The requirements for soft smart wearable garments can be conflicting: stretchable, entirely conformable to the body, designed ergonomically and esthetically pleasing, small size and weight, flexible, washable, robust, unobtrusive, reliable, and durable (Yang and Yin, 2021). Person identification is an integral part of smart gait devices due to the need for customized user experience, which introduces the need to preserve users’ privacy. Additionally, continuous, seamless user authentication is vital to prevent malicious attacks on users’ medical records without the burden of frequently entering a personal identification number (PIN) (Xu et al., 2021). Finally, other requirements include water-proof capability, mechanical durability, and connectivity with other smart devices and environments depending on the specific use (Zou et al., 2020). To accommodate these requirements, the main focus of the research is on sensing technology. Sensors for wearables are IMUs, capacitive sensors (Lan et al., 2020), KEH (Xu et al., 2017; Xu et al., 2018), (Ma et al., 2018), solar cells (Sandhu et al., 2021), resistive sensors, stretchable conductive micro fluids (Low et al., 2020), and TENGs (Zhang et al., 2020g).

There is generally a trade-off between the complexity of sensing technology in smart gait devices and the accuracy of the intelligent task they perform (Khademi et al., 2019). Depending on the task and granted sufficient accuracy, one can stop the chase for performance and focus on patient comfort (Di Nardo et al., 2020). A multi-object optimization (MOO) technique is implemented (Khademi et al., 2019) to navigate this trade-off: it selects an optimal feature subset that maximizes accuracy while minimizing sensing hardware. Fusing different bio-signals in hBCI systems for gait applications improves classification accuracy and the number of control commands. Still, it introduces the problem of channel configuration, information transfer rate, and temporal synchronization between the modalities (Khan et al., 2021).

SG devices will be an integral part of smart cities, smart buildings, smart homes, smart transportation, smart factories, energy grids, and e-Healthcare and are poised for tremendous growth in the future as IoT in the 5G framework will facilitate better and faster inter-connectivity between the devices (Chettri and Bera, 2019), end-to-end deep learning algorithms will provide real-time intelligence, and sensing technology will be embedded more comfortably into our everyday objects and clothing.

### 7.2 Smart Homes

A smart home environment is defined as one that acquires and applies knowledge about its residents and their physical surroundings to improve their experience in that setting (See Table 13). The smart home sensors, the wearable sensors, and the classifying algorithms in the CASAS smart home (Cook et al., 2015) serve to perform health monitoring, early detection of disease, health care, and treatment. The smart home integrates wearable sensing technology, AI technology, and sensor fusion technology to automatically control home appliances via a gesture recognition algorithm, to turn lights on and off via an indoor positioning algorithm, and to set the alarm off via a fire detection algorithm (Hsu et al., 2017). With a focus on XAI, a numerical score of anomaly level is output on the monitor along with natural language explanations (Khodabandehloo et al., 2021). The privacy and security of health data are major concerns of smart homes. With the recent advancements in blockchain technology and its wider acceptance, a possible solution is to build smart blockchain networks to securely store and share health data (Taralunga and Florea, 2021) (Cernian et al., 2020).

**TABLE 13:** Smart home applications.

Reference	AI Algorithm	Data Acquisition	Purpose
Borelli et al. (2019)	AI algorithms are built into smart objects	Wall light for indoor localization.	This paper provides a complete description of the HABITAT project regarding methodology, architecture, design, and smart objects development
	The smart armchair and the smart belt perform activity recognition algorithms.	Armchair for sitting posture monitoring.The belt for movement information.	
	The wall light sends input to a fall detection algorithm	The Wall panel and mobile devices are the user interface	
Hsu et al. (2017)	3D gesture recognition: 95.3% using PNN and 10-fold CV.Pedestrian navigation: distance and positioning accuracies were 0.22 and 3.36% of the total distance traveled in the indoor environment.Home safety and fire detection: classification rate 98.81%.	Wearable IMU on wrist tracks hand gesture and on feet walking data and energy managementEnvironmental sensors.Experimental smart home testbed. Web camera. Multisensory circuit module for home safety and fire detection	Design and implementation of a smart home system that integrates wearable intelligent technology, artificial intelligence, and sensor fusion technology to complete these tasks:Automated household appliance control.Smart energy management.
	PNN, DTW, SVM, LDA, PDR, PCA-PNN.	Fire detection and home safety
Cook et al. (2015)	DT, NBC, RF, SVM, Ada/DT, Ada/RF are tried out, and Ada/DT provides the best classification accuracy.	CASAS smart home and wearable sensors.Analysis 1: *N* = 75 PD = 25 HC = 50.	In home health monitoring for early detection of changes associated with PD and MCI and evaluation of treatment
	PCA is used to reduce features k-Means clustering and random resampling are used to add features in smaller (individual activities) datasets	Analysis 2: *N* = 52, PD ann No MCI = 16, PD with MCI = 9, HC = 18, MCI and no PD = 9Subjects perform IADL tasks in a CASAS smart home testbed	

Legend: Pseudo-odometry (P-O), Adaptive Boosting (Ada), Probabilistic Neural Network (PNN), Mild Cognitive Impairment (MCI), Instrumental ADLs (IADLs), Dynamic Bayesian Network (DBN).

Of interest in this discussion is the project HABITAT (Home Assistance Based on the Internet of Things for the Autonomy of Everybody) that had only four key AI applications at the time this paper was written: an indoor localization system, smart armchair, smart belt, and a wall panel and mobile devices as the user interface (Borelli et al., 2019). The smart home embeds smart objects into objects of everyday life. The belt, which assesses body movement, is of interest to this study since it captures postural transitions and gait biometric data. The AI modules follow the Event Calculus (EC) modelling approach, which makes it easy to define the properties of the system. The system is expandable, more smart devices can be incorporated into it, and is an example of what is possible when activities and objects of our daily lives become “smart.” In summary, AI algorithms take care of the “smart” part of the home. Instead of being a passive recipient of care, the patient becomes an active agent of an intelligent health ecosystem (Najafi and Mishra, 2021).

SG extends beyond the context of smart home and will play a major role in the future smart cities (Lozano Domínguez and Mateo Sanguino Tde, 2021), smart factories (Pech et al., 2021), smart retail stores (Zhang et al., 2019a), smart rehabilitation labs (Sessoms et al., 2015), and smart devices (Lozano Domínguez and Mateo Sanguino Tde, 2021)

## 8 Animation and Virtual Environments

For this review, we consider studies requiring and using gait data and implementing an ML algorithm to create animated characters for movies, video games, and virtual reality environments. These studies include research in gait modeling, motion reconstruction, and character control. (See Table 14)

**TABLE 14:** Animation and virtual environments.

Reference	AI Algorithm/Characteristics	Data Acquisition/Inputs	Task
Feigl et al. (2020)	THR, COR, SVM and BiLSTM, tested	*N* = 6, head-mounted accelerometer data	Motion reconstruction
	- COR has the best accuracy for real-time VR applications (low delay)		Gait phase detection
Bergamin et al. (2019)	DReCon: motion matching and deep RL	Unstructured motion data from mocap	Real-time physics-based character control for video games
	- responsive to user demands, natural-looking. Trained on flat terrain		
Peng et al. (2018b)	OpenPose/HMR and DRL	Simulated character model and YouTube video clip	Learning dynamic physics-based character controllers from video clips
	- Learning from inexpensive video clips, robust		
Peng et al. (2018a)	DeepMimic: DRL	Character model, kinematic reference motion from video clip	Physics-based character controllers from video clips
	- Diverse skills/terrains/morphologies, realistic response to perturbations		
Huang et al. (2018)	SMPL body model and BiLSTM	6 IMUs	3D human pose reconstruction from a sparse set of IMUs
	- Useful when camera-based data is not available due to occlusion, fast motion, etc		
Holden et al. (2016)	CAE	CMU Motion Capture Database ³	Unsupervised learning of a human motion manifold
	- Capable of fixing corrupt data, filling in missing data, motion interpolation along the manifold, and motion comparison		
Huang et al. (2015)	SMG and part-based Laplacian deformation	Three 4DPC datasets ^4^	A data-driven approach for animating 4DPC character models
	- Simultaneously captures both motion and appearance for video-like quality		
Ding and Fan, (2015)	Multilayer JGPMs/topologically constrained GPLVMs	CMU Motion Capture Database + Simulated data	Human gait modeling
	- diversity of walking styles, motion interpolation, reconstruction, and filtering		
Alvarez-Alvarez et al. (2012)	FFSM with automatic learning of the fuzzy KB by GA	*N* = 20	Human gait modeling
	- Fuzzy states and transitions are still defined by experts, interpretable, generalizes well for each person’s gait	Accelerometer attached to the belt	

Legend: Threshold Based Method (THR), Pearson Correlation-based Method (COR), Data-Driven Responsive Control (DReCon), Human Mesh Recovery (HMR), Deep Deterministic Policy Gradient (DDPG), Skinned Multi-Person Linear (SMPL) as in (Loper et al., 2015), 4D Performance Capture (4DPC), Surface Motion Graphs (SMGs), Carnegie Mellon University (CMU), Joint Gait-Pose Manifolds (JGPMs), Fuzzy Finite State Machines (FFSM), Knowledge Base (KB).

Datasets: ³ http://mocap.cs.cmu.edu/ ^4^ http://cvssp.org/cvssp3d.

In Virtual Reality (VR) applications, the virtual character is controlled real-time by the user’s behavior. The task of the AI algorithm is to correctly recognize the user’s gait phases, with no delay, for a good real-time visual representation of the avatar’s motion. In video games, deep reinforcement learning is the algorithm of choice where the policy controls the action at each step. The policy is defined carefully to positively reward the desired action at each step, such as maintaining balance, tracking pose and orientation, alignment, mimicking the reference motion, surviving perturbations, and negatively rewarding actions such as falls. The avatars must be real-looking, preserving both the motion extracted from the skeleton features and the shape (Loper et al., 2014), (Huang et al., 2015).

Responsiveness to user demand, quality of visual representation, robustness to different walking styles and terrains, system runtime performance, adaptation to disturbances, maintaining balance, and retargeting to different morphologies are some of the key goals of these systems. Usually, motion data is extracted from expensive high-quality multi-camera motion capture systems. Authors have studied the possibility of retrieving high-quality representations from low-cost video clips recorded with pervasive monocular videos such as YouTube clips (Peng et al., 2018b) and reconstructing human pose from inertial measurements, in the case when direct-line-of-sight camera recording is not available due to occlusion for instance (Huang et al., 2018).

## 9 Discussion and Future Trends

In this study, we reviewed different applications of the smart gait with a focus on the various tasks artificial intelligence algorithms perform across many industries and disciplines, such as health and wellness, security, forensics, and energy management. We further identify four emerging trends in the SG research: 1) population-wide scale health data will be available and empower end-to-end automatic, ubiquitous, and continuous deep learning approaches for big data-driven intelligent systems, 2) Fast-growing innovations in other technologies such as cloud computing, smart textiles, blockchain, and 5G will offer new opportunities and pose new challenges for SG systems and demand fast congruent growth, 3) SG systems will need to address concerns about user privacy, safety, comfort, and experience captured by the paradigm, “human-in-the-loop” (Sedighi Maman et al., 2020); these will be enforced by human rights advocates and regulatory bodies, and 4) the need for AI in health applications to benefit from the fast, low-cost, and accuracy of “black box” intelligent systems while still being transparent and understandable, as captured by the paradigm of XAI. SG is a valuable tool in kinetic and kinematic analysis, disease monitoring, diagnosis, and rehabilitation, sports performance, fall risk assessment, detection and prevention, gait-based biometrics for person identification, re-identification and continuous automatic authentication, age and gender recognition, physical skill and mobility assessment, fitness tracking, gait modeling and simulation, crowd monitoring and anomaly detection, human pose estimation, indoor tracking, and localization. SG is often integrated with other smart systems that utilize biomedical signals such as electrocardiograms, body parameters such as temperature, blood pressure, respiration rate, energy expenditure, and heart rate, and environmental signals such as room temperature and humidity. SG is often part of a smart IoT framework embedded with sensors, monitoring devices, and AI-enabled actuators that are all connected and in continuous communication. SG is everywhere, in our smart devices, smart homes, classrooms, cars, stores, cities, and energy grids. Smart Gait research will continue to grow fast in the future and will benefit from advancements in other technologies such as sensors, blockchain, IoT, textiles, 5G, cloud computing, and big data. These new technologies will also pose new demands and offer new opportunities for smart gait research. SG research will continue to address user privacy issues, security of health data, patient comfort, worker safety in the workplace, enhanced user experience, fatigue monitoring and injury prevention in sports.

We also identify these three needs for the SG research community: 1) inter-disciplinarity 2) the need for SG to become an organized field of study with specific definitions and good practices in place, and 3) the need for open competition and collaboration. Looking into the future, digital technologies such as smartwatches, headbands, and smartphones will be exploited to collect population-wide scale data to facilitate health monitoring and early detection of various diseases. One aspect of such systems is the multimodality of the data (Frey et al., 2019), (Abeysekara et al., 2020). Their integrated approach will demand cross-disciplinary research and collaborations. The gait research teams will need to include experts across many disciplines. We see the collaborative culture becoming more prevalent in the future both, within the SG community and between the SG community and the larger research community across many fields. With the recent development of programming languages and the open research community around them on Github, Kaggle, and other online and app-based forums, it is possible and important that the Smart Gait research community is open and the datasets, AI code, and suggested strategies for improvement are available for future collaborative work. For instance, some authors have made the data and the ML toolbox public and available for other researchers (Baghdadi et al., 2021) and (Horst et al., 2019). Wherever such open and collaborative efforts have flourished in the past, the results have been outstanding. Computer vision studies have seen tremendous results by reducing the cost of entry to new research and encouraging collaboration and competition towards a goal. The authors of this paper are excited to see similar growth in the SG research community.

## 10 Conclusion

The utilization of AI in gait analysis is a growing field. In this paper, we refer to it as the Smart Gait. It is compellingly multi-disciplinary, drawing from cutting-edge research in multiple mathematical and engineering fields, and it continues to welcome new approaches and new data-capturing methods. With the constant advances in wearable sensors technology, cloud computing, and new advances in ML, the progress is formidable. Coupled with the growing realization of real-world applications such as non-invasive person identification, person re-identification, intruder detection, medical diagnosis and treatment, advanced fall detection, etc., will keep the demand for new gait detection and analysis methods high and keep AI at the forefront of the research. The field should only grow and expand in scope over the next 10–15 years. From smart home devices to smart grids and smart cities, AI is here to stay, and it will become very pervasive. AI-driven gait-based systems will take the shape of a chair you sit on, shoes you wear, a mat you exercise on. The smart door of your home will open as it recognizes your walk via a continuous SG authentication system, and the phone will lock itself in the hands of the thief as the abnormal gait is recognized. A crowd density algorithm will warn you of high Covid-19 risk when you enter a store.

With the growing demand and promising results, accessibility remains a limitation. ML is a tool, and as such, it should be in the hands of those who need it; early detection of PD in the hands of the clinician, monitoring of treatment in the hands of the caregiver, occupancy sensing in the hands of a family who cares about energy management in their home, indoor tracking and localization in the hands of the police in your town police station, kinematic analysis in the hands of a competing athlete. For ML systems to be more accessible, the technology will need to become easy to understand and implement. It must become less expensive and more scalable. XAI is a trend that will continue. The experts in related fields and the general population will continue to become more AI savvy. In a not-too-distant future, knowing how to use an open-source Python library or write your line or two of code in R will be as common a task as writing an email today. With that will come privacy concerns, ethical concerns, and a need to adjust our laws and regulations as a society.
